# Women’s experience following orthodontic treatment with conventional fixed appliances and clear aligners: A qualitative study

**DOI:** 10.1371/journal.pone.0359709

**Published:** 2026-11-02

**Authors:** Nicolle San Nicolas Dubrull Lia Lucarelli, Gabriela Gonçalves Innocente, Pedro Augusto Thiene Leme, Luana Paz Sampaio, Luiz Gonzaga Gandini Junior, Silvia Amélia Scudeler Vedovello, Lucas Arrais Campos, Carolina Carmo de Menezes

**Affiliations:** 1 Department of Morphology and Pediatric Clinic, São Paulo State University-UNESP, School of Dentistry, Araraquara, Brazil; 2 Department of Health Sciences and Pediatric Dentistry, Orthodontics, Piracicaba School of Dentistry, Universidade Estadual de Campinas (UNICAMP), Piracicaba, São Paulo, Brazil; 3 Institute of Dentistry, School of Medicine, Faculty of Health Sciences, University of Eastern Finland, Kuopio, Finland; UCLA School of Dentistry, UNITED STATES OF AMERICA

## Abstract

This qualitative study aimed to explore the experiences of female patients following completion of orthodontic treatment with conventional fixed appliances and clear aligners. It used a clinical qualitative design with, semi-structured interviews featuring open-ended questions. Participants were recruited from private orthodontic practices, and the sample size was determined by data saturation. The final group included six patients treated with conventional fixed appliances and nine patients treated with clear aligners. Data were transcribed verbatim and analyzed using qualitative content analysis with peer validation of the categories. Seven categories emerged among patients treated with conventional fixed appliances: pain, discomfort, and hygiene difficulties; daily adaptations and restrictions; confidence in the professional; treatment duration; insecurity or embarrassment; desire to improve appearance; and satisfaction with treatment outcomes*.* For patients treated with clear aligners, seven categories were identified: practicality, comfort and hygiene; pain and discomfort related to use; treatment duration; willingness to recommend aligners; desire to improve appearance and appliance discretion; recommendation by the professional; and satisfaction. The findings show that, although patients with fixed appliances faced several challenges during treatment, aesthetic improvement was the main reason for seeking care, and overall satisfaction with the results was high. Patients who chose clear aligners aimed to avoid common limitations of fixed appliances and emphasized benefits related to comfort, convenience, oral hygiene, and discretion. These results highlight the importance of qualitative methods for understanding patient perspectives and can guide clinical communication and shared decision-making in adult orthodontic treatment.

## Introduction

Increasingly, adult patients seek orthodontic treatment to improve esthetics, oral function, and psychosocial well-being [1,2]. This growing demand has been accompanied by a preference for less visible, more comfortable, and easier-to-clean orthodontic appliances [2]. During orthodontic treatment, patients may experience changes in speech, eating habits, and social interactions, as well as varying degrees of pain [3].

Quantitative studies have extensively examined patients’ perceptions and satisfaction with conventional fixed appliances and clear aligners [2,4–8]. These studies indicate that satisfaction with orthodontic treatment is influenced by factors such as physical pain, functional limitations, psychological discomfort, physical disability, and esthetic changes [2,8]. Systematic synthesis of patient-reported outcomes (PROs) indicates generally high satisfaction after orthodontic treatment but reveals meaningful variation according to appliance type, clinician–patient communication, and treatment context [9]. Available qualitative research has explored patients’ perceptions of conventional fixed appliances among both adolescents and adults [3,10–13]. Among adolescents, studies report positive esthetic effects of orthodontic appliances [11], alongside behavioral changes affecting general and oral health, psychosocial factors [12], self-perception of malocclusion, treatment motivations, and expectations regarding outcomes [10]. In adult patients, qualitative findings emphasize the role of communication, the dental team, and the perceived influence of treatment on daily life [13]. In addition, a qualitative meta-synthesis suggests that orthodontic treatment positively affects patients’ self-esteem, social interactions, and esthetics [3].

Qualitative methodologies are particularly valuable for exploring patients’ experiences with orthodontic treatment, as they allow a deeper understanding of treatment expectations, personal perspectives, and satisfaction with outcomes, thereby informing clinical decision-making and treatment goals [2,3,14]. By focusing on patients’ narratives, qualitative research offers rich and detailed insight into how individuals interpret and make sense of their treatment experiences [3]. These approaches provide access to patients’ opinions, perceptions, beliefs, and attitudes that may not be fully captured through standardized quantitative measures [10].

Recent evidence has linked the use of clear aligners to esthetic satisfaction and improvements in oral health–related quality of life, while also indicating a temporary increase in pain at the beginning of treatment that may negatively affect these outcomes [14]. However, this evidence is largely derived from quantitative designs, including scoping and systematic reviews, which primarily focus on treatment outcomes and patient satisfaction scores rather than on patients’ lived experiences throughout the therapeutic process.To date, qualitative investigations specifically exploring adult patients’ post-treatment experiences with clear aligners—and directly comparing them with conventional fixed appliances—remain scarce. Little is known about how adult women interpret and attribute meaning to different aspects of orthodontic treatment, such as discomfort, esthetics, social interactions, and daily life demands, once therapy has been completed.

Therefore, the present study adopted a qualitative approach, guided by the Clinical-Qualitative Method [15], to explore the meanings and importance that adult female patients assign to their experiences after completing orthodontic treatment with conventional fixed appliances and clear aligners.

## Materials and methods

The Clinical-Qualitative Method (CQM) was employed to assess patients’ experiences at the conclusion of orthodontic treatment. The study design was adopted for its flexibility and emphasis on understanding subjective meanings in clinical contexts [15]. This approach allows iterative refinement of the interview process through interaction between interviewer and participant, facilitating exploration of experiences while remaining responsive to emerging themes [16,17]. Data were collected using semi-structured interviews with open-ended questions, enabling participants to describe their experiences in their own words while ensuring coverage of key topics relevant to orthodontic treatment [18].

Qualitative research seeks to understand the meanings that specific groups ascribe to phenomena experienced in their natural contexts [19]. Accordingly, such studies are conducted in real-life settings rather than under controlled experimental conditions, allowing participants’ perspectives to emerge with minimal researcher interference [16]. This approach entails particular considerations regarding sample composition, analytic procedures, and the transferability—rather than statistical generalization—of findings, all of which guided the methodological choices of the present study [16].

### Sampling and recruitment

Eligible participants were female patients aged 17 years and older who had completed orthodontic treatment with either conventional fixed appliances or clear aligners and were willing to participate after providing written informed consent. Orthodontic treatment had been completed at least six weeks before the interview, with a maximum interval ranging from approximately 1.5 to 33 months prior to data collection [13]. The decision to include only female patients was defined a priori. This methodological choice was informed by the need to ensure sample homogeneity, thereby allowing exploration of shared experiential patterns. Patients were excluded if they declined to participate or had hearing impairment, significant speech or learning difficulties, craniofacial syndromes, cleft lip and/or palate, or a history of orthognathic surgery [13,20,21].

Participants were recruited from the private practices of four orthodontists with both clinical and academic experience, all located in the same city in São Paulo, Brazil. Orthodontists identified eligible patients who had completed treatment within the defined time frame and informed them about the study. Potential participants were subsequently contacted by the first author via telephone, during which the study objectives were explained, and an invitation to participate was extended. Interviews were scheduled according to participants’ availability after initial agreement. Formal written informed consent was obtained prior to data collection.

An acculturation phase comprising two initial interviews was conducted prior to formal data collection. During this phase, the semi-structured interview guide was tested and refined, and the process also served as methodological training for the interviewer. Data from these interviews were excluded from the final analysis.

All interviews were conducted by the first author via Google Meet and were audio-recorded with participants’ permission. Interviews were conducted individually and were not repeated. Interview duration ranged from approximately 5 to 16 minutes (median ≈ 9.6 minutes; interquartile range ≈ 6.5–13.6 minutes). Brief field notes were recorded after each interview.

After conducting a certain number of interviews, the sample was closed using the theoretical data saturation technique. This method allowed the researcher to identify the point at which responses started to repeat or no longer provided meaningful insights, thereby ending the data collection stage. Saturation was assessed collaboratively by the research team. Upon reaching saturation, the sample was closed, resulting in a final sample of fifteen participants. Data collection took place between January and February 2025.

### Ethical approval

After approval by the Research Ethics Committee for Human Subjects at the Araraquara School of Dentistry, São Paulo State University “Júlio de Mesquita Filho,” Araraquara, SP, Brazil (CAAE: 74900123.4.0000.5416), this qualitative study was conducted in a virtual environment. Individuals were invited to participate in the study, and those who agreed signed the Informed Consent Form and were included in the study sample.

### Qualitative data collection

Semi-structured interviews were conducted to explore patients’ experiences with orthodontic treatment after its completion. An initial interview guide was developed with input from three orthodontists experienced in qualitative research and was informed by previous studies in the field [10,13,22,23]. The guide was designed to encourage open reflection on treatment experiences while ensuring that key topics relevant to orthodontic therapy were addressed.

All interviews were conducted by the first author, a female orthodontist and trained researcher with prior experience in qualitative interviewing. All interviews were audio-recorded, transcribed, and analyzed. The interviewer guided participants throughout the conversation, clarifying questions when necessary and encouraging detailed responses. The interviewer had no prior relationship with the participants before recruitment, and participants were informed only of the study's general purpose. Although the interviewer is an orthodontist, which may shape the interpretation of clinically related accounts, reflexive awareness was maintained throughout data collection and analysis through analytic memos, and interpretations were continuously discussed with the research team during category development to challenge assumptions and minimize potential researcher bias.

Each participant was interviewed only once, as repeated interviews were considered unnecessary given the focused, post-treatment nature of the inquiry. Although interview durations were relatively brief, the interviews addressed a specific, clearly delimited phenomenon rather than a broad life history, allowing participants to speak directly to the aspects of interest about post-treatment perceptions. Follow-up questions were used whenever clarification or further elaboration was needed, and participants generally illustrated their accounts with concrete examples from their treatment experience. The achievement of theoretical data saturation further indicated that the interviews provided sufficient depth to address the study objectives.

The interviews followed the semi-directed interview technique with open-ended questions, as proposed by Turato (2003) [15]. This approach begins with a brief reintroduction between interviewer and interviewee, during which the objectives of the study are reiterated, and the informed consent procedures are reviewed. Sociodemographic information was collected prior to the start of the interview. Throughout the interview, participants were encouraged to speak freely and to associate ideas spontaneously, with minimal interruption by the interviewer [15,17,24].

Interviews were conducted online via Google Meet and audio-recorded with participants’ permission. To protect confidentiality, participants were instructed to join the interview from a private setting using personal devices, and only audio recordings were stored. No video or screen recordings were made. Audio files and transcripts were stored on password-protected institutional servers accessible only to the research team.

Interviews began with an open-ended question, the first being: *“I would like you to tell me about your experience with the fixed appliance (or) aligner.”* Depending on the flow of the conversation, the interviewer used followup questions to make sure the study objectives were covered. These additional questions included: *“What was it like for you to brush your teeth while wearing the appliance?”*, *“Did you notice any differences during meals?”*, and *“Did you ever feel embarrassed about wearing the appliance?”*

Two additional open-ended questions were then posed: *“What led you to choose this treatment?”* and *“Are you satisfied with the treatment results?”* The interview ended with the final prompt: *“Would you like to add any comments or talk about something that was not asked?”*

During the interviews, the interviewer periodically summarized participants’ statements and invited confirmation or clarification, which functioned as in-interview clarification of participants’ meanings. Field notes were recorded after each interview to document contextual information and initial impressions.

### Data analysis

The data were analyzed using Qualitative Content Analysis, following a seven-step process [15,19,25,26]. In the first step, all interviews were transcribed verbatim by the first author, who also produced initial analytic notes during transcription. The second step, transcripts were listened to and read repeatedly to promote immersion in the data, which the principal researcher also carried out. During the third step, two researchers independently reviewed the transcripts and recorded preliminary comments and impressions based on the re-reading and discussed emerging findings. Next, in the fourth step, these researchers refined the categories and selected excerpts from the interviews. In the fifth step, three researchers presented and discussed the emerging categories, enabling peer debriefing and critical reflection. In the sixth step, the first author grouped coded excerpts into themes representing shared meanings and subsequently consolidated these themes into final categories.

In the seventh and final step, the category definitions were reviewed by the research team through consensus discussion, referred to here as peer validation. The analysis generated 39 condensed meaning units (17 for participants treated with fixed appliances and 22 for those treated with clear aligners), which were grouped into initial codes and subsequently consolidated into seven final analytic categories for each treatment modality (S2 File). The COREQ checklist is provided as S1 File. Given the modest sample size and the emphasis on interpretive depth, qualitative data were managed using structured tables and text-editing software rather than specialized qualitative analysis software.^26^

For participants treated with conventional fixed appliances, preliminary analysis after the first four interviews suggested that theoretical data saturation might have been reached. Two additional interviews were subsequently conducted to confirm this assumption. No new meaningfully distinct themes emerged during these interviews, supporting the conclusion that saturation had been achieved, as illustrated in Table 1.

**Table 1:** Evidence of sample saturation indicated by the appearance of categories related to the research questions (patients treated with conventional fixed appliances).

Initial Categories – Participants	E1	E2	E3	E4	E5	E6
Discomfort/pain during the first days of adjustment	x	x	x	x		x
Negative experience with fixed appliance use	x			x	x	
Adaptation and routine restrictions during appliance use	x	x		x	x	x
Confidence in the professional	x	x				x
Difficulty with oral hygiene	x	x	x	x	x	x
Convenience of treatment with clear aligners	x			x		x
Esthetic concerns	x	x	x	x	x	x
Satisfaction with treatment outcome	x	x	x	x	x	x
Professional recommendation	x	x	x	x		
Insecurity/Embarrassment	x				x	x
Treatment duration		x	x	x	x	x
**New categories**	10	1	0	0	0	0

X indicates that the category was present in the corresponding interview. The ‘New categories’ row indicates how many new categories emerged in each interview. No additional new categories emerged after E2.

For participants treated with clear aligners, preliminary analysis after six interviews suggested that data saturation was approaching. Three additional interviews were conducted, during which no new themes relevant to the research objectives emerged. These findings confirmed saturation within this group, as summarized in Table 2.

**Table 2:** Evidence of sample saturation indicated by the appearance of categories related to the research questions (patients treated with clear aligners).

Initial Categories – Participants	E1	E2	E3	E4	E5	E6	E7	E8	E9
Convenience and Comfort		x	x	x	x	x		x	
Ease of oral hygiene	x	x	x	x	x	x	x	x	x
Discomfort when changing trays	x		x	x		x	x		
Family members’ experience with orthodontic treatment	x	x	x						x
Esthetic concern (discreet?)	x			x	x				x
Satisfaction with treatment outcome	x	x	x	x		x	x	x	x
Professional recommendation	x	x					x		
Treatment duration		x	x					x	x
Speech/diction			x			x	x		x
Would recommend aligners		x				x	x		
**New categories**	6	3	1	0	0	0	0	0	0

X indicates that the category was present in the corresponding interview. The ‘New categories’ row indicates how many new categories emerged in each interview. No additional new categories emerged after interview E3.

Because the saturation grid was constructed from the same interviews used to declare saturation, it represents a within-sample, post-hoc description of the analytic process rather than an independent external validation of saturation.

## Results

Participants described their experiences with orthodontic treatment using conventional fixed appliances and clear aligners during individual interviews. Analysis yielded seven final categories for each treatment modality, reflecting shared meanings related to treatment experience, motivation, and outcomes. Illustrative excerpts are presented below, with participant identification indicated by the letter “P” followed by a number. Participants were classified according to the appliance modality corresponding to the treatment experience explored during the interview; although some participants — including retreatment cases — reported previous or concurrent experience with the other modality, the analysis focused on the modality that constituted the primary subject of the interview. Participants’ sociodemographic characteristics are summarized in Table 3.

**Table 3:** Sociodemographic characteristics of the participants.

Participant	P1	P2	P3	P4	P5	P6	P7	P8	P9	P10	P11	P12	P13	P14	P15
**Age**	17	45	40	45	31	48	52	69	44	38	41	45	39	30	44
**Education**	HSD	CD	CD	CD	CD	CD	CD	CD	CD	CD	CD	CD	CD	CD	CD
**Treatment**	AL	AL	AL	AL	AL	FA	AL	AL	AL	FA	FA	AL	FA	FA	FA
**Residence**	AQA	AQA	AQA	AQA	USA	Matão	Teresina	AQA	AQA	AQA	Taquaritinga	AQA	AQA	AQA	AQA
**Treatment completion year**	2022	2022	2024	2024	2024	2024	2024	2024	2023	2024	2023	2022	2024	2024	2024
**Interview duration (min:sec)**	15:54	15:03	14:56	08:41	06:11	13:22	07:19	13:53	07:18	09:39	05:28	06:29	11:52	05:51	11:27

P = participant; HSD = high school diploma; CD = college degree; AL = clear aligner; FA = fixed appliance; AQA = Araraquara. Interview duration is reported in minutes and seconds.

### Experience with orthodontic treatment using conventional fixed appliances

Regarding treatment with conventional fixed appliances, participants described experiences related to discomfort, daily adaptations, professional trust, treatment duration, motivation for treatment, and satisfaction with outcomes. Eleven initial categories were identified and subsequently consolidated into seven categories, which demonstrated internal homogeneity and mutual heterogeneity (Table 4).

**Table 4:** Categories assigned to participants who received treatment with conventional fixed appliances.

Categories related to the experience of using this type of appliance
**1st category** – Pain, discomfort, and hygiene
**2nd category** – Adaptations and restrictions
**3rd category** – Confidence in the professional
**4th category** – Treatment duration
**5th category** – Insecurity/Embarrassment
**Category related to the motivation for seeking orthodontic treatment with this type of appliance**
**6th category** – Desire to improve appearance
**Category related to the outcome of orthodontic treatment with this type of appliance**
**7th category** – Satisfaction

#### 1st Category – Pain, discomfort and hygiene.

This category reflects participants’ reports of discomfort, particularly during the initial phase of treatment and during appliance adjustments.

“Fixed braces are uncomfortable at first, you know? Until you get used to them again, they hurt the inside of your mouth a lot.” (P6)

According to the participants, fixed appliances caused significant pain at the start of treatment because of the adaptation period. Pain was also reported during appliance adjustments.

“I found it uncomfortable... every time it gets activated, there’s some pain, right? When it’s activated... I found hygiene very complicated. Later, in day-to-day life (...) cleaning, flossing.” (P1)

The experience reported by participants using conventional fixed appliances was not pleasant:

“So, like... because it would stress me out... I had a lot of neck pain, so when they tightened the braces, I had to go to physical therapy. So, it wasn’t one of the best experiences.” (P3)

Difficulty using dental floss and properly brushing teeth with fixed appliances was also a common complaint among participants:

“I used a lot of force, because you get the impression that nothing is getting clean, and I only realized this after I had the braces removed—my toothbrush wasn’t wearing the same way anymore. I had to replace it much more often.” (P5)

“I found out I didn’t know how to brush... I was just brushing the appliance, you know? I couldn’t brush my gums, and using dental floss was really difficult.” (P2)

#### 2nd Category – Adaptations and restrictions.

Participants reported needing to adjust their eating routines when using fixed appliances.

“So, when eating, you had to be careful, avoid eating certain types of food... things that would stick to the braces and be hard to remove... a lot of restrictions, so to speak.” (P6)

Many foods could not be consumed or required extra caution when eating:

“Oh, eating becomes terrible. For the next two days, it feels like all your teeth are going to fall out (...) everything has to be cut into small pieces. Everything changes. You have to adapt your entire routine, you know? Brushing, eating. Then there are things you think twice about eating because cleaning afterwards will be a hassle.” (P4)

Some participants also compared the experience of wearing conventional fixed appliances to clear aligners, noting that the adaptations and restrictions differ between the two:

“...the discomfort of eating in public, having to deal with all that... if it were the kind of appliance they have nowadays, right? Invisalign, you just take it out, eat, brush your teeth, and put it back in.” (P6)

“The doctor explained everything to me about what had to be done after getting the braces. But even so, it’s really complicated (...) for me, it wasn’t a great experience, but I see the final result and it was worth it. So, when I started, I don’t even know if Invisalign existed yet... I think now everything’s more practical, easier.” (P1)

#### 3rd Category – Confidence in the professional.

The treatment experience also relies on the orthodontist, as feelings of safety, patient care, and clear explanations about the therapy are essential for achieving good treatment outcomes.

“At that other clinic, I think the professional wasn’t very well prepared (...) she said that if I closed all the spaces, my lips would sink in. So, this esthetic part affects you (...) I had two experiences, right? One with a not-so-prepared professional—I felt she was insecure—and then with Dr. [name], it was totally different.” (P2)

“We are very well cared for. It’s a very humanized experience. They are excellent, both as people and as professionals.” (P1)

#### 4th Category – Treatment duration.

The duration of treatment with conventional fixed appliances was also a key concern mentioned by participants. A longer-than-expected treatment period or the discomfort from extended appliance use was reported:

“The dentist said it would be for a certain period, and it ended up being much longer.” (P3)

“After my third molar came in, I had to remove it, and my teeth shifted again (...) at first, I liked it—I thought it was great and wanted to do it again. I hated it. It was terrible, just awful. I couldn’t stand wearing the braces anymore. No way.” (P5)

#### 5th Category – Insecurity and embarrassment.

Another recurring theme in the interviews was the insecurity linked to wearing fixed appliances in social situations. Many participants covered their mouths when smiling or avoided smiling in photos.

“I did feel some discomfort, yes, especially when taking a photo... you don’t feel comfortable smiling... there’s a bit of insecurity when it comes to smiling.” (P1)

Shame was also evident in social food-related situations.

“Situations where there was food along with a social gathering, you know? You had to talk and eat at the same time—I think those were the most awkward moments... you spend the whole time trying to hide your mouth.” (P5)

### Categories related to the motivation for seeking orthodontic treatment with this type of appliance

#### 6th Category – Desire to improve appearance.

Improving the look of the smile was the main reason for getting orthodontic treatment. In many cases, this involved retreatment.

“It was mostly for esthetic reasons. The first time I had braces; I had a gap on the bottom row that was almost the size of a finger. I had managed to fix that, but then when they extracted two teeth, I was left with those extraction gaps.” (P2)

In other cases, treatment was driven by family pressure because of dissatisfaction with the patient’s smile.

“I wore braces for a long time. I had gotten used to the gaps and wasn’t really bothered by them anymore, but my husband was.” (P2)

In some cases, orthodontic treatment was necessary before performing a desired esthetic procedure.

“I went to a dentist... to do some cosmetic work. Then he said, ‘for us to do the esthetic part you want, we first need to align your teeth.’” (P1)

In some interviews, patients pursued orthodontic treatment based on recommendations from another dentist, often for aesthetic reasons.

“So, actually, when I started... I didn’t even know... I mean, I had an idea that I needed to align my teeth, right? A dentist I went to once mentioned it to me.” (P1)

“Before getting the implant, I had to adjust my bite to place the implant in the best position.” (P3)

Retreatment cases also fall into this category, as professionals identified the need for another round of orthodontic therapy.

“I’ve worn braces twice (...) but the first time I wore them at the right age (...) if you wait too long, it gets worse (...) the first time was just to align my teeth, but even then I was already a candidate, because I had—what’s it called?—dental protrusion. I had to have four teeth extracted.” (P4)

### Categories related to the outcome of orthodontic treatment with this type of appliance

#### 7th Category – Satisfaction.

Although participants reported many negatives, satisfaction with the treatment's final result was a common theme.

“It got much better. I couldn’t close my mouth—my dental arch was really protruded—so the result was very good.” (P4)

Some participants mentioned the need to finish the process with additional esthetic procedures, such as teeth whitening, implants, or resin restorations.

“I was satisfied. I still need to do the esthetic part. But I really liked the result (...) the doctor explained exactly how much my lip would retract and everything.” (P2)

### Experience with orthodontic treatment using clear aligners

Regarding treatment with clear aligners, various aspects were linked to the participants’ experiences. Based on the results, 10 initial pre-categories were identified and later consolidated into 7 broader categories: 4 related to the experience of using this type of appliance, 2 related to the motivation for seeking orthodontic treatment with clear aligners, and 1 related to the treatment outcome. These categories demonstrated internal homogeneity and mutual heterogeneity, as shown in Table 5.

**Table 5:** Categories assigned to participants who received treatment with clear aligners.

**Categories related to the experience of using this type of appliance**
**1st category** – Practicality, comfort, and hygiene
**2nd category** – Pain and discomfort related to use
**3rd category** – Treatment duration
**4th category** – Willingness to recommend clear aligners
**Categories related to the motivation for seeking orthodontic treatment with this type of appliance**
**5th category** – Desire to improve appearance and appliance discretion
**6th category** – Recommendation by the professional
**Category related to the outcome of orthodontic treatment with this type of appliance**
**7th category** – Satisfaction

#### 1st Category – Practicality, comfort and hygiene.

Participants frequently highlighted practicality and comfort during interviews as important factors influencing their choice of clear aligners:

“It’s an appliance you can remove to eat, and that was a huge issue for me—I had never used braces before because of the metal parts, right? The aligner gave me the opportunity to go through treatment without that discomfort.” (P4)

“I had never worn braces before, right? It was my first experience, so I don’t have anything to compare it to (...) I loved it, I found it practical and hygienic, because you take it out to eat and you can clean it before putting it back in.” (P8)

Additionally, all participants mentioned the ease of maintaining hygiene when using clear aligners participants:

“Perfect. You place it in the right product to clean it, and brushing is easy—you can floss, which you can’t do with the other appliance, because it gets stuck. So it was simply very comfortable to use.” (P4)

#### 2nd Category – Pain and discomfort related to use.

Another common topic was the discomfort linked to changing the aligner trays.

“When I first started, I felt a bit of pain, you know? But it went away over time. Every time the tray tightened, I felt pain, but that also passed. And it really boosted my self-esteem (...) it was something I’d wanted to do for a while to improve myself (...) align my smile. And for me it was great. The experience was very good.” (P1)

“(...) it gives you a lot of freedom, right? One thing I didn’t like was that if you pay attention, it fills with saliva. Little bubbles form (...) people notice (...) I could feel it when I switched trays—on the first day I’d feel it. When it tightens and I go to eat, things feel sensitive. But only on the first day.” (P2)

“Sometimes when I put it in... I could feel things moving a little, a slight discomfort, but it only lasted a day (...) in the first few days of switching trays, you’d feel it... I wouldn’t even call it pain—more like discomfort.” (P7)

Speech difficulties were also noted during the initial phase of treatment:

“I really liked it. I thought it was very effective. I thought it was faster than fixed braces, which I had worn previously. And I found it very hygienic (...) in the beginning I had some trouble with my tongue, but it went back to normal.” (P9)

“(...) being able to take it out to eat, then brush and put it back in. So I had no problems (...) in the beginning it affected my speech, you know? Until I got used to it—about two weeks.” (P3)

#### 3rd Category – Treatment duration.

Several participants mentioned the duration of treatment with clear aligners, perceiving it as quicker than with fixed appliances.

“I really liked it. I thought it was effective. I thought it was faster. I thought it was discreet. I thought it was hygienic. In fact, my daughter even got them too.” (P9)

“My treatment was really fast. It lasted ten months. And I followed everything properly (...) the doctor said, ‘if all my patients did everything like this, treatments would be much quicker.’” (P8)

“It seemed faster than braces. And I can see it actually was—because I started at the same time as a friend. She got fixed braces, I got aligners. I already finished mine and she’s still wearing hers.” (P3)

Another point mentioned was that some patients opted not to undergo a refinement phase even if the treatment was not fully completed according to the professional’s plan:

“I was already tired. The first phase lasted 16 months. And it didn’t quite get to where it should have... so after that, I had to wear them for another 13... but there was still a little left. The dentist wanted things to come together a bit more... but I didn’t want to continue.” (P2)

#### 4th Category – Willingness to recommend clear aligners.

The recommendation of aligner treatment was a theme brought up during interviews:

“The only thing I didn’t like was talking with them (...) something about it bothered me. But aside from that, no complaints. I totally recommend it.” (P6)

“...if I had to recommend this treatment to everyone and not use the other type anymore.” (P7)

“If someone asked me whether I’d recommend it, I would. But the person has to be disciplined. Very disciplined.” (P2)

### Categories related to the motivation for seeking orthodontic treatment with this type of appliance

#### 5th Category – Desire to Improve appearance and appliance discretion.

In the case of clear aligners, the motivation was often connected to enhancing the appearance of the smile and choosing a discreet treatment option.

“It was great for my self-esteem. It was something I had wanted to do for a long time—to improve myself. To align my smile.” (P1)

“I thought it was discreet.” (P9)

“The aligner gave me the chance to go through treatment without that discomfort... without it looking like I was wearing braces... it’s practically invisible.” (P4)

“Esthetically, it’s much better than the traditional type of braces.” (P5)

#### 6th Category – Professional recommendation.

In some interviews, participants reported seeking aligner treatment after a referral from another dentist.

“I went to a dentist for a routine cleaning... I had a lot of tartar on my teeth... she told me, ‘you have a lot of tartar because your mouth is open... if you go through orthodontic treatment and close your mouth, that will reduce the tartar.’ So I went ahead and looked into it.” (P2)

“I’ve been told I needed orthodontics since I was little, and back then only traditional braces existed, right? And since I have a tendency to get cavities... I kept imagining myself wearing those traditional braces, and when I tried Invisalign, it was so much easier.” (P7)

### Category related to the outcome of orthodontic treatment with this type of appliance

#### 7th Category – Satisfaction.

Most participants reported satisfaction with the final outcome of their orthodontic treatment using clear aligners.

“Very satisfied.” (P9)

“It exceeded my expectations, because when I started using it, I was a bit skeptical. I kept thinking, ‘Can this little piece of plastic really move my teeth into place?’” (P4)

Few participants elaborated on the outcome itself, often summarizing their impression by simply stating they were satisfied with the result.

## Discussion

The study of the meanings and interpretations attributed by patients has been extensively explored across various fields of public health, including dentistry.^3^ In this context, exploring orthodontic treatment experiences through a qualitative approach provides valuable insight into how patients interpret discomfort, esthetics, daily challenges, and treatment outcomes beyond what can be captured by quantitative measures alone.

The present study focused on adult female patients; a methodological decision was made a priori based on evidence that perceptions of orthodontic treatment, esthetics, and social experiences may vary by gender [13]. Focusing on women enabled a more nuanced understanding of how orthodontic treatment is integrated into daily life, self-image, and social contexts. Given that the subjective experience of body image, health care, and clinical interactions often carries distinct symbolic meanings across genders, the use of an all-female sample provided a more cohesive symbolic universe for the interpretation of latent meanings. The age range of participants (17–69 years) allowed exploration of experiences across adulthood while maintaining a relatively homogeneous group in terms of life stage [13,22]. In qualitative research, sampling is purposeful, involving the deliberate selection of individuals who have experienced or are knowledgeable about the phenomenon of interest. The sample typically includes participants who represent the characteristics of a specific subpopulation, and the number of participants is determined in the field rather than preset, with data collection continuing until theoretical saturation was achieved [19]. Therefore, this study examined the experiences of adult female patients, including six who used conventional fixed appliances and nine who used clear aligners. All participants received orthodontic treatment in private practices and had completed secondary education. Participants were interviewed between 1.5 and 33 months after treatment completion, which enabled reflection on the active treatment phase while minimizing immediate recall bias [13].

Experiences associated with conventional fixed appliances were largely characterized by pain, discomfort, daily adaptations, and concerns about treatment duration. These findings are consistent with previous reports describing functional limitations, pain, speech alterations, dietary restrictions, and social challenges during fixed appliance therapy [3,27]. It is well established that fixed appliances are associated with higher levels of discomfort, particularly during the initial stages of treatment, and may lead to prolonged pain when compared with clear aligners [28]. Rather than functioning as an isolated dimension of the experience, this discomfort appeared to be interpreted by participants in relation to the perceived quality of the clinical relationship: when communication and reassurance from the orthodontist were adequate, transient pain seemed to be more readily absorbed into an acceptable treatment narrative, whereas its absence appeared to intensify dissatisfaction.

In addition to pain, difficulties related to oral hygiene were frequently reported by participants treated with fixed appliances. The presence of brackets, wires, and auxiliary components complicates brushing and flossing, often increasing the risk of plaque accumulation and patient dissatisfaction [29]. Given the extended duration commonly associated with fixed appliance therapy, these challenges underscore the importance of comprehensive oral hygiene instruction and continuous professional support to promote adherence and maintain oral health throughout treatment. This hygiene-related burden did not appear to stand as an independent category in participants’ accounts. Rather, it seemed to become more tolerable when patients perceived their orthodontist as attentive and available, suggesting that professional support operates less as a peripheral courtesy and more as a mediating factor shaping how burdensome hygiene demands were ultimately experienced.

In contrast, patients treated with clear aligners consistently emphasized practicality, comfort, and ease of hygiene as key advantages influencing their treatment choice. Although direct comparisons with prior qualitative studies are limited by the scarcity of similar methodologies, quantitative evidence supports these perceptions, demonstrating lower chewing discomfort, reduced impact on self-esteem, improved sleep comfort, and higher overall satisfaction among aligner users [2,7,30]. Notably, the practicality attributed to aligners was frequently discussed alongside participants’ confidence in having chosen the appropriate professional and treatment modality, suggesting that perceived convenience and trust in that initial decision were mutually reinforcing ones.

Discomfort among aligner users was primarily associated with tray changes and transient speech difficulties during the initial phase of treatment. Existing evidence suggests that such speech alterations are typically temporary, with most patients adapting within one to two months [31,32]. Despite ongoing technological advances, aligners may still be accompanied by unrealistic expectations regarding pain reduction and treatment duration, reinforcing the importance of patient education and realistic counseling during treatment planning. As with fixed appliances participants tended to situate this discomfort within a broader narrative of trust in the gradual, professionally guided progression of trays, which appeared to lessen its perceived significance over time.

Professional recommendation emerged as a relevant factor influencing treatment choice for both appliance types. Participants frequently reported that clinical indications related to malocclusion and referrals from dental professionals guided their decisions [33]. Previous studies also highlight the influence of the dental environment, professional communication, and interdisciplinary referrals on patients’ overall treatment experience [3]. This finding suggests that trust in the professional is often established early, prior to appliance-specific experiences, and may relate to the way in which discomfort, hygiene demands, and esthetic concerns are appraised throughout the course of treatment.

The social aspect of wearing fixed appliances was highly important to participants, as many expressed insecurity and embarrassment about the visibility of their braces. At the same time, the desire for aesthetic improvement was a common motivation for seeking treatment. It is well established that the most common expectations for orthodontic treatment include the ability to smile confidently in public, improved eating, and better dental alignment. These findings align with the existing literature, which shows that expectations for orthodontic treatment often include improved self-confidence, enhanced esthetics, and better social interactions [12,34]. Participants seemed to weigh visible discomfort against anticipated esthetic gain, with trust in the professional's judgment about the necessity and expected duration of treatment serving as an interpretive frame that made this trade-off acceptable.

Overall satisfaction with treatment outcomes was high among participants treated with both fixed appliances and clear aligners. Importantly, patients may evaluate treatment success differently from clinicians, reinforcing the growing emphasis on patient-centered care and the inclusion of psychological and experiential factors when assessing orthodontic outcomes [35]. Taken together, these findings suggest that satisfaction did not result simply from the balance of positive and negative experiences, but from how aesthetic improvement and trust in the professional relationship allowed participants to reinterpret earlier discomfort as a worthwhile trade-off [3,34]. In this sense, trust appeared to function as an interpretive bridge across otherwise disparate categories as pain, hygiene burden, and esthetic concern, allowing participants to integrate them into a largely positive narrative of treatment, rather than evaluating each category independently.

For patients who used clear aligners, esthetics was associated not only with an improved smile but also with the appliance's discreet appearance. Aligners offer significant quality-of-life benefits compared to fixed appliances, especially in social interactions, self-esteem, and overall satisfaction with oral health. However, despite these esthetic advantages, some participants noted that aligners are not entirely invisible, and the buildup of saliva within the trays caused discomfort in social settings [8]. Even this residual discomfort, however, was described by several participants as a manageable inconvenience once weighed against their confidence in the aligner system and in the orthodontist's guidance, again pointing to trust as a factor moderating the perceived severity of discomfort.

Trust in the orthodontist and the quality of professional communication were particularly salient among patients treated with fixed appliances. Clear explanations perceived technical competence, and a welcoming clinical environment positively influenced treatment experiences and acceptance of discomfort. These findings reinforce existing evidence that effective communication is a cornerstone of patient satisfaction and cooperation during orthodontic treatment [13]. This suggests that trust functioned not merely as a feature of the patient–professional relationship, but as a mechanism through which participants reframed the pain, hygiene difficulties, and daily adaptations described earlier as acceptable, transitory costs of treatment rather than as purely negative experiences [13,34].

Considered together with the patterns described above, this mechanism was observed consistently across the pain, hygiene, and esthetic categories, indicating that trust operate as a transversal interpretive axis running through participants’ accounts, mediating the extent to which different forms of discomfort were tolerated and ultimately reframed as acceptable.

Trust in the professional relationship seems to operate as a mediating axis linking otherwise categories of experience. Physical discomfort, hygiene-related burden, and esthetic concern were seldom evaluated in isolation. Rather, participants appeared to interpret them through the lens of their confidence in the orthodontist's competence, communication, and clinical judgment, which allowed transient negative experiences to be reframed as acceptable costs within a broader satisfactory treatment trajectory.

Despite the insights provided by this study, limitations inherent to the study design should be acknowledged. The sample consisted of female patients treated in private orthodontic practices. Consistent with the principles of qualitative research using the Clinical-Qualitative Method, the goal is not to achieve statistical representativeness, but to explore the depth of lived experiences among patients who have completed a specific orthodontic treatment. Consequently, the findings may have limited applicability to male patients, adolescents, or individuals treated in public or lower-resource settings. Furthermore, participants were recruited through the clinics where they had received orthodontic treatment, which introduces the possibility of self-selection and social desirability bias: women who were more satisfied with their treatment outcomes may have been more willing to participate, while those with less positive experiences may have declined the invitation or provided more favorable accounts, potentially contributing to the predominantly positive perceptions observed. The timing of post-treatment interviews is an additional methodological consideration: interviews were conducted 1.5–33 months after treatment to allow participants to reflect on their orthodontic experience and to provide more stable, consolidated meanings, consistent with the Clinical-Qualitative Method, although variability in this interval may have influenced recall. Taken together, these factors should be considered when interpreting the findings, which reflect the experiences of this specific group of women rather than of orthodontic patients in general; future studies employing different methodological approaches are warranted to test emerging hypotheses and support broader generalization of the data.

Notwithstanding these limitations, this qualitative study offers valuable insights into how adult female patients perceive and assess their orthodontic treatment experiences after finishing therapy. By emphasizing patient-reported experiences with both fixed appliances and clear aligners, the findings enhance a more patient-centered understanding of orthodontic care and may guide clinical communication, expectation management, and shared decision-making in adult orthodontics.

## Conclusion

According to this study, the categories reported by female patients who received treatment with conventional fixed appliances primarily reported challenges such as discomfort, oral hygiene difficulties, adaptations, restrictions, and feelings of embarrassment. The main motivation for choosing this type of treatment was to enhance aesthetics.

In contrast, patients who chose clear aligners were primarily motivated by the desire to avoid issues often linked to fixed appliances. During treatment, aligners were seen as more comfortable, easier to handle, better for oral hygiene, and more discreet in appearance.

The qualitative methodology enabled a thorough examination of these experiences, showing that although patients faced challenges during treatment with fixed appliances, they trusted their orthodontists, were kept informed about all treatment stages, and were satisfied with the final results.

## Supporting information

S1 File File_COREQ_updated. COREQ (Consolidated Criteria for Reporting Qualitative Research) checklist. Completed 32-item COREQ checklist indicating the manuscript pages where each reporting criterion for qualitative research is addressed.
(DOCX)

S2 File Coding framework and analytic categories derived from participant interviews. Anonymized condensed meaning units and corresponding analytic categories derived from interview transcripts of participants treated with fixed appliances and clear aligners.
(DOCX)
