# Supplementary material for: Women’s experience following orthodontic treatment with conventional fixed appliances and clear aligners: A qualitative study
Source: PLoS One. 2026 Nov 2;21(11):e0359709. doi: 10.1371/journal.pone.0359709 (PMC13726917; doi:10.1371/journal.pone.0359709)
Supplement: Supplementary file 2 [file pone.0359709.s002.docx]

| **Participant ID** | **Treatment Type** | **Code (Condensed Meaning Unit)** | **Analytic Category** |
| --- | --- | --- | --- |
| E1 | FA | Pain during activation | Pain, discomfort, and hygiene |
| E2 | FA | Difficulty flossing | Pain, discomfort, and hygiene |
| E5 | FA | Difficulty brushing teeth | Pain, discomfort, and hygiene |
| E6 | FA | Initial oral discomfort | Pain, discomfort, and hygiene |
| E4 | FA | Food restrictions | Adaptations and restrictions |
| E6 | FA | Avoiding certain foods | Adaptations and restrictions |
| E4 | FA | Changes in daily routine | Adaptations and restrictions |
| E2 | FA | Trust in orthodontist | Confidence in the professional |
| E1 | FA | Feeling well cared for | Confidence in the professional |
| E3 | FA | Treatment longer than expected | Treatment duration |
| E5 | FA | Fatigue with prolonged treatment | Treatment duration |
| E1 | FA | Embarrassment in photos | Insecurity and embarrassment |
| E5 | FA | Social discomfort while eating | Insecurity and embarrassment |
| E2 | FA | Desire to improve smile | Desire to improve appearance |
| E1 | FA | Orthodontics before esthetic procedure | Desire to improve appearance |
| E4 | FA | Satisfaction with final result | Satisfaction |
| E2 | FA | Need for complementary esthetics | Satisfaction |
| E4 | AL | Removable for meals | Practicality, comfort, and hygiene |
| E8 | AL | Easy to clean and hygienic | Practicality, comfort, and hygiene |
| E4 | AL | Facilitates oral hygiene | Practicality, comfort, and hygiene |
| E1 | AL | Pain during tray change | Pain and discomfort related to use |
| E2 | AL | Sensitivity on first day | Pain and discomfort related to use |
| E7 | AL | Mild temporary discomfort | Pain and discomfort related to use |
| E9 | AL | Temporary speech difficulty | Pain and discomfort related to use |
| E3 | AL | Adaptation period for speech | Pain and discomfort related to use |
| E9 | AL | Perceived faster treatment | Treatment duration |
| E8 | AL | Short treatment duration | Treatment duration |
| E3 | AL | Faster than fixed appliances | Treatment duration |
| E2 | AL | Did not complete refinement phase | Treatment duration |
| E6 | AL | Would recommend treatment | Willingness to recommend aligners |
| E7 | AL | Strong recommendation | Willingness to recommend aligners |
| E2 | AL | Requires discipline | Willingness to recommend aligners |
| E1 | AL | Improvement in self-esteem | Desire to improve appearance and discretion |
| E9 | AL | Discreet appearance | Desire to improve appearance and discretion |
| E4 | AL | Nearly invisible appliance | Desire to improve appearance and discretion |
| E2 | AL | Referral by dentist | Professional recommendation |
| E7 | AL | Recommendation due to oral condition | Professional recommendation |
| E9 | AL | Very satisfied with results | Satisfaction |
| E4 | AL | Results exceeded expectations | Satisfaction |
|  |  |  |  |

**Notes**

- FA = Fixed appliance
- AL = Clear aligner
- Codes represent condensed meaning units derived from interview transcripts
- Analytic categories correspond to the final categories presented in the Results section
- Only anonymized, non-identifiable excerpts are included
- This dataset reflects the analytic structure used to support the study findings
